# Conflict of Interest Policies at French Medical Schools: Starting from the Bottom

**DOI:** 10.1371/journal.pone.0168258

**Published:** 2017-01-09

**Authors:** Paul Scheffer, Christian Guy-Coichard, David Outh-Gauer, Zoéline Calet-Froissart, Mathilde Boursier, Barbara Mintzes, Jean-Sébastien Borde

**Affiliations:** 1 Sciences of Education Department, Paris 8 University, Saint-Denis France; 2 Saint-Antoine Hospital, Paris, France; 3 Faculty of Medicine Purpan, Toulouse 3 University, Toulouse, France; 4 Faculty of Medicine Aix-Marseille, Marseille, France; 5 Faculty of Medicine Rennes1, Rennes, France; 6 Faculty of Pharmacy, Charles Perkins Centre, The University of Sydney, Sydney, Australia; 7 Saintonge Hospital, Saintes, France; State University of New York, Oswego, UNITED STATES

## Abstract

**Background:**

Medical faculties have a role in ensuring that their students are protected from undue commercial influence during their training, and are educated about professional-industry interactions. In North America, many medical faculties have introduced more stringent conflict of interest (COI) policies during the last decade. We asked whether similar steps had been taken in France. We hypothesized that such policies may have been introduced following a 2009–2010 drug safety scandal (benfluorex, Mediator) in which COIs in medicine received prominent press attention.

**Methods:**

We searched the websites of all 37 French Faculties of Medicine in May 2015 for COI policies and curriculum, using standardized keyword searches. We also surveyed all deans of medicine on institutional COI policies and curriculum, based on criteria developed in similar US and Canadian surveys. Personal contacts were also consulted. We calculated a summary score per faculty based on 13 criteria. [range 0–26; higher scores denoting stronger policies]

**Results:**

In total, we found that 9/37 (24%) of French medical schools had either introduced related curriculum or implemented a COI-related policy. Of these, only 1 (2.5%) had restrictive policies for any category. No official COI policies were found at any of the schools. However, at 2 (5%), informal policies were reported. The maximum score per faculty was 5/26, with 28 (76%) scoring 0.

**Conclusion:**

This is the first survey in France to examine COI policies at medical faculties. We found little evidence that protection of medical students from undue commercial influence is a priority, either through institutional policies or education. This is despite national transparency legislation on industry financing of health professionals and limits on gifts. The French National Medical Students Association (ANEMF) has called for more attention to COI in medical education; our results strongly support such a call.

## Introduction

Several initiatives have been undertaken in the last decade to protect medical students from the influence of the pharmaceutical and medical devices industries, and to better educate future doctors about potential commercial influences on their medical practice. Among them, conflict of interest (COI) policies of medical schools have received special attention. Since 2007, the American Medical Student Association (AMSA) has published an annual scorecard ranking American medical schools on their COI policies. AMSA used 14 criteria, based on the research literature on the subject [1]. Mason and Tattersall published a study on Australian medical schools in 2009 based on similar criteria [2], and Shnier et al. did the same for Canada in 2013 [3]. Limited regulation of conflict of interest was reported in both countries, but there were also some encouraging measures: in Canada, the Association of Faculties of Medicine of Canada (AFMC) has voted to support the 2008 recommendations by the Association of American Medical Colleges (AAMC) to better manage, and when necessary, prohibit relationships between academics and industry that can lead to COI and undermine professional standards [3]. AAMC represents 145 accredited American faculties, 17 accredited Canadian faculties and over 400 teaching hospitals. In the United States overall, two thirds of American medical schools received an A or B on AMSA scorecards in 2015, which corresponds to exemplary or moderate scores. This reflects major shifts in policy in less than a decade. In 2007 nearly all medical schools had received a F, the worst grade possible [1].

These initiatives reflect the need for medical education to be based on the best medical science available, protected from commercial biases. However, conflicts of interest are still common in the classroom. In many medical faculties, students are taught by faculty members who have ties with the industry. They may have received research grants, be members of companies' speakers' bureaus or advisory committees [4]. This leads to a conflict of interest, where financial partnerships of faculty may influence, or reasonably appear to influence, their professional medical opinions, with potential effects on the integrity of what they teach to medical students [5,6]. The behavior of teachers and the information they provide is very important as they have a large influence on medical students as role models [7].

The situation is similar in many ways in France. Following the benfluorex (Mediator^®^) safety scandal in 2009–2010, tougher regulations governing conflicts of interest were introduced [8,9]. Benfluorex is closely related to fenfluramine and dexfenfluramine, appetite suppressants that were removed from the market in Europe and North America in the late 1990s. Benfluorex remained on the French market until 2009 and is estimated to have led to over 3000 hospitalizations and around 1300 deaths during its 33 years on the French market [10]. Conflicts of interest were identified as having played a substantial role in the slowness of regulatory action, leading to the introduction of stronger regulation [8,9]. Since March 2016, French law requires faculty members to disclose their competing interests in the classroom, following a position taken by the European Council [11,12]. This is consistent with the wishes of French students to know the COI of their lecturers, as expressed in a 2012 survey [13].

Apart from influences in the classroom, relationships with industry can also take the form of advertising published in medical students’ textbooks and information in industry-sponsored educational materials. Until the 2009 to 2010 health scandals in France, two pharmaceutical firms, including the producer of benfluorex (Mediator^®^) Servier, sponsored and/or organized a preparatory session for the National Classifying Exams (ECN), which ranks the students after their 6^th^ year of study, and determines their future area of medical practice. The majority of preclinical and clinical students are also frequently exposed to pharmaceutical industry influences through drug representatives, gifts, meals, advertising in textbooks or by participating in sponsored educational programs [14]. International research shows that medical students are vulnerable to these influences, although most believe they are personally immune [15–20]. A recent study indicated that French medical students, similarly, had many contacts but believed themselves not to be influenced. In this survey, most of the students reported a lack of education on COI [13]. However, several initiatives indicate that students do have an interest in improved education in this field, including a 2015 statement by the National Association of the French Medical Students (ANEMF) publicly supporting the present study on COI in medical education [21].

In 2013, the first research results were published indicating the effects of the introduction of stricter COI policies in medical schools in the US, indicating effects on medical residents’ prescribing practices. In two studies comparing graduates of programs with more or less stringent policies, those who studied or pursued residencies in institutions with more stringent policies and who had a longer experience of the policy had the best prescribing results [22,23]. These research results show that implementation of stricter COI policies does have a beneficial effect on practice. On the contrary, when there are no or only weak policies restricting industry influence, medical students are directly and indirectly exposed to commercial strategies aiming to influence their way of thinking about medical issues and their prescribing practices. This is all the more important as the attitudes and behaviors formed in medical school have been shown to persist into professional life [2]. Given these research results in the US, we decided to carry out a study on COI policies of all medical faculties in France.

## Methods

As no patients are involved in our study, and it relates to questions concerning policies at respondents’ institutions, rather than personal information, the Comité pour la Protection des Personnes (Comittee for human protection) Saint-Antoine, has written us that ethics approval was not required for our research.

Our methodology was based on the criteria used in the AMSA Scorecard and Shnier et al. [1,3].

A list of the 37 French medical schools was obtained from the website of l'ISNCCA (Intersyndicat National des Chefs de Clinique Assistants des hôpitaux de ville de facultés–National Inter Trade-union of the Assistant Senior Registrars of hospitals and faculties) [24].

We searched the website of each medical school in May 2015 to find policies related to COI or documents interpreting policies using the French terms: « conflits d'intérêts » (conflict of interest), « liens d'intérêts » (competing interests), « industrie pharmaceutique » (pharmaceutical industry), « laboratoire pharmaceutique » (pharmaceutical firm), « charte » (charter), et « règlement intérieur » (internal rules). The name of each policy and the latest date of adoption or the date of the policy’s most recent review were recorded.

A registered letter was sent to each office of the Dean of medicine to inform them of the study. The letter explained the study’s purpose, the criteria for which we required documentation, requested confirmation that these criteria were relevant for each particular school, and confirmed that we had not missed or inadvertently left out any existing policies. The letter mentioned also the fact that our search of the medical school’s website research had uncovered no policy, as this was the case for all 37 medical schools. This allowed the representative of the Dean’s office to send a correction should we have overlooked an existing policy, and to send us any draft policy not yet implemented.

The offices of the Dean were informed that we were only interested in publicly available policies and while respondents’ names would be kept confidential, the medical schools and their policies would be identified in any subsequent publication. We enclosed two letters of support, one from Barbara Mintzes, co-author of the Shnier et al. study which evaluated the policies of Canadian medical schools [3] and editor of the teaching manual *Understanding and Responding to the pharmaceutical promotion* [25], and the other one from the president of ANEMF (Association Nationale des Etudiants en Médecine de France–National Association of the French Medical Students).

This was followed by a maximum of five reminders to non-respondents. If we did not receive an initial response, a reminder email was sent in July 2015. In September 2015, we sent an email with our initial results, encouraging the offices of the Deans to send us any corrections or new information that should be included, with a reminder email in October 2015. We then sent our final results to the deans by email in November 2015, providing them the opportunity to send corrections or modifications, with a reminder in December 2015.

In addition to websites and contacting the offices of the Deans of medical faculties, we sought additional information via personal contacts and experts in the field.

We did not search for and request policies from affiliated teaching hospitals because they are not under the authority of the dean of the medical school and our study concerns the first six years of medical training, before the start of internship.

A scoring system was adapted to the French context from the criteria used by Shnier et al. [3]. We excluded two criteria, drug samples and industry support for scholarships from our list. Samples are not allowed in France [26], and and there is no industry scholarship support for medical students because medical training in France occurs in public medical schools.

In France, medical faculties can receive funding from companies through the apprenticeship tax. This tax is imposed by the State and is principally used to finance professional education. It may affect the decisions and independence of a medical faculty. Pharmaceutical companies also provide educational support to medical residents to assist them with publication of scientific articles. This can play an important role in a doctor’s career and is worth taking into consideration. Lastly, much medical education takes place in teaching hospitals. We decided to follow AMSA’s example [1] and to include a separate criterion concerning a medical school’s promotion of adoption of COI policies by affiliated teaching hospitals (criterion 13 below).

The first 10 criteria below are the same as 10 of Shnier et al.’s criteria [3]. We added the last three criteria (#11 to 13 inclusive).

1. Gifts (including meals).
2. Consulting relationships (excluding scientific research and speaking).
3. Industry-funded speaking relationships/speakers’ bureaus.
4. Honoraria from pharmaceutical industry.
5. Ghostwriting.
6. Disclosure.
7. Industry Sales Representatives.
8. On-site Education Activities.
9. Compensation for travel or Attendance at Off-site Lectures & Meetings.
10. Medical school curriculum (or other documentation of educational objectives/course content).
11. Pharmaceutical industry funding of the medical school.
12. Industry educational support of residents for publication of scientific articles.
13. Medical school activities to promote COI policies in affiliated teaching hospitals.

Similarly to the approach taken by the AMSA Scorecard and Shnier et al., we also considered whether there were procedures in place for monitoring and enforcement. We assessed two aspects of monitoring and enforcement: 1) whether there was clearly a party responsible for general oversight to ensure compliance, and 2) whether there were sanctions for noncompliance. Each of these enforcement measures was scored either “yes” or “no.” We did not attempt to identify if policies had been violated or to grade the severity of sanctions.

We graded each criterion on a scale of 0 to 2 where 0 means no policy or a permissive policy, 1 a moderate policy and 2 a restrictive policy (See S1 File for detailed scoring criteria for each category). Two reviewers independently scored each medical school’s policies, and the entire group reviewed the scoring, with any disagreements resolved through discussion. Similarly to Shnier et al. [3], we summed the scores of the first thirteen individual categories for each school to come up with a global score, with a range of 0 to 26 points. Each category was weighted equally.

## Results

The 37 medical faculties listed by ISNCCA on their website include 35 located in France and 2 located in overseas territories. All of these medical schools have a website. See S1 Table for a list of the medical faculties and their websites.

### Internet searches

We were unable to retrieve any COI policy or mention of COI in the curriculum on the websites of the 37 medical faculties, including those located in overseas French territories (Outre-Mer), despite carrying out an extensive keyword search, as described above. We retrieved information on coursework related to COI in the curriculum of two faculties, ***Lyon Sud Medical School*** and ***Paris 5—René Descartes Medical School***.

The ***Lyon Sud Medical School*** offers an optional educational module on “medicine and the pharmaceutical industry” which includes a component of “permitting students to have a global vision and critique the pharmaceutical industry”, but this course is taught in large part by pharmaceutical industry representatives.

The ***Paris 5 –René Descartes Medical School*** offers two courses for medical students called “managements of financial ties and conflicts of interest”.

### Mail and email survey of representatives of the Dean’s offices of Medical Schools

Out of 37 offices of the Dean contacted, we received three responses from ***Lyon Est***, ***Angers*** and ***Toulouse Purpan***.

The office of the Dean of the ***Lyon Est Medical School*** answered that the medical school has no conflict of interest to declare, as it receives no private or industrial financing. It clarifies that the medical school does not accept the apprenticeship tax. As described above, this tax on pharmaceutical and device companies is used to provide funding for professional education. ***Lyon Est*** is also opposed to the practice exams organized by pharmaceutical firms. Students’ conduct concerning financial ties or contacts belongs to their individual responsibility, with no requirement to report to the medical school. The curriculum does not include any training on financial links or conflicts of interest, based on information available on-line.

The office of the Dean of the ***Angers Medical School*** stated that the medical school has no policy to prevent or manage conflicts of interest. The ***Angers Medical School*** receives the apprenticeship tax provided by the pharmaceutical and medical device industry. It offers an optional course on pharmaceutical industry influences for fourth year medical students. Events sponsored by pharmaceutical companies are allowed in the medical school if not labeled “Continuing Medical Education”.

In the opinion of the representative of the Dean’s office of the ***Angers Medical School***, it is unlikely that faculty members would accept explicit control of additional activities linked with pharmaceutical industry. This person stressed that given the current financial situation, medical schools cannot refuse private sector financing.

The office of the Dean of the ***Toulouse Purpan Medical School*** did not consider our enquiry to be legitimate and provided no information, as medical schools can be audited by broader French public administration: IGAS (Inspection Générale des Affaires Sociales—General Inspection of Social Affairs) or IGAENR (Inspection Générale de l’Administration de l’Education Nationale—General Inspection of Administration of National Education).

### Data collected through personal contacts with instructors

Through personal contacts, we obtained information on courses that cover COI at seven medical schools. These were at ***Lyon Est*, *Aix-Marseille*, *Strasbourg*, *Toulouse Purpan*, *Rennes 1*, *Paris 5*, *and Paris 7*.** These courses represent isolated initiatives by teachers who recognize the importance of COI issues. They are electives (optional courses, not within core curriculum). For this reason, all of them received a score of one for the curriculum category, according to our grading system (see S1 File).

### Medical schools’ scores

To the best of our knowledge, by June 2016, no French medical school had developed written policies concerning conflict of interest or interactions with the pharmaceutical industry.

Table 1 summarises the results of our Internet search and correspondence with the medical schools. Of the 37 medical faculties, we found nine (24%) with either a COI policy of some sort or inclusion of COI in the curriculum. These medical faculties included COI on the curriculum but in a limited way. Two of them (5%) had COI policies, ***Lyon Est*** and ***Angers***, reflecting the information provided by the deans as described above. Only one faculty (2,5%), ***Lyon Est***, had restrictive policies for some categories (On-site education, and funding by pharmaceutical industry). This same medical school achieved the best score recorded with a total of 5 points, out of a possible maximum of 26 points. Twenty-eight medical schools (75%) scored 0.

**Table 1:** Conflict of interest policy scores for each French medical faculty (n = 9)*.

School	Strength of policy				Enforcement	
	No/permissive	Moderate	Restrictive	Total**	Party	Sanction
	Score = 0	Score = 1	Score = 2			
Lyon Est	• Sales rep. • Honoraria • Compensation • Disclosure • Ghostwriting • Gifts • Promotion • Publication • Consulting • Speaking	• Curriculum	• On-site • Funding	(5)	No	No
Angers	• Sales rep. • Honoraria • Compensation • Disclosure • Ghostwriting • Gifts • Funding • Promotion • Publication	• On-site • Consulting • Speaking • Curriculum	-	(4)	No	No
Aix-Marseille	• Sales rep. • Honoraria • Compensation • Disclosure • Ghostwriting • Gifts • Funding • Promotion • Publication • On-site • Consulting • Speaking	• Curriculum	-	(1)	No	No
Lyon Sud	• Sales rep. • Honoraria • Compensation • Disclosure • Ghostwriting • Gifts • Funding • Promotion • Publication • On-site • Consulting • Speaking	• Curriculum	-	(1)	No	No
Paris 5	• Sales rep. • Honoraria • Compensation • Disclosure • Ghostwriting • Gifts • Funding • Promotion • Publication • On-site • Consulting • Speaking	• Curriculum	-	(1)	No	No
Paris 7	• Sales rep. • Honoraria • Compensation • Disclosure • Ghostwriting • Gifts • Funding • Promotion • Publication • On-site • Consulting • • Speaking	• Curriculum	-	(1)	No	No
Rennes 1	• Sales rep. • Honoraria • Compensation • Disclosure • Ghostwriting • Gifts • Funding • Promotion • Publication • On-site • Consulting • Speaking	• Curriculum	-	(1)	No	No
Strasbourg	• Sales rep. • Honoraria • Compensation • Disclosure • Ghostwriting • Gifts • Funding • Promotion • Publication • On-site • Consulting • Speaking	• Curriculum		(1)	No	No
Toulouse Purpan	• Sales rep. • Honoraria • Compensation • Disclosure • Ghostwriting • Gifts • Funding • Promotion • Publication • On-site • Consulting • Speaking	• Curriculum	-	(1)	No	No

* An additional 28 French medical faculties had no information on conflict of interest policies affecting medical students on their websites, and failed to reply to our request for information (See S1 Table for the list of all French medical schools).

** The highest score possible is 26. All scores are in parentheses as the policies were described by the representatives of the Dean’s offices of medicine, but without written documentation or mention on the faculty’s website, and the curriculum is optional.

## Discussion

Our study results indicate little attention to the issue of COI in French medical education, and few explicit COI policies. We found that only 2 of the 37 French medical faculties had any COI policies, but that even for these two medical faculties, these policies were not available as written documents nor made public on the medical faculty’s websites.

Only three Dean’s offices chose to answer to our enquiry, despite the multiple letters sent between June 2015 and January 2016. Our results suggest that there is little attention to COI by French medical schools, unlike the situation in North America where both the AAMC and the AFMC (Association of Faculties of Medicine in Canada) have adopted positions on COI and invited their member faculties to adopt the relevant changes. The extent to which the AAMC and AFMC have followed up to verify and encourage implementation remains unclear, however. The 2010 AFMC report includes a statement that lack of progress on these questions could have a negative impact on accreditation [27]. This may have had an effect on the decision to implement. Laval University, in Quebec, provides an example of how difficult and fragile the implementation of these measures may be. In 2015, Laval revised its 2009 conflicts of interest policy, citing lack of adequate public financing, which led to a number of instructors in the medical faculty to seek more financial support from pharmaceutical companies and to consider the companies to be ‘partners’. This type of collaboration is seen in a positive light in the 2015 policy, with the suggestion that it is preferable for students to have contact with pharmaceutical companies in order to learn how to manage them rather than to avoid such contacts [28]. This is inconsistent with a number of recommendations on COI and medical education, including the American Medical Students’ Association (AMSA)’s position [29]. While the 2015 Laval policy does bring in a committee to oversee situations in which a potential conflict of interest is identified, it appears to have also loosened restrictions on COI as compared with Laval’s 2009 policy.

In 2005, medical faculties in Norway introduced a collective agreement excluding the pharmaceutical industry from medical education, no longer allowing industry-sponsored events on campuses. This is the only comprehensive initiative of the sort in Europe. A survey of Norwegian medical students carried out in 2009–2010 found that most students reported relatively little interaction with the industry but less than a third were aware that their medical school had guidelines restricting interactions [7]. We were unable to find any research on the longer-term outcomes of the 2005 measures such as effects on prescribing practices.

Had we only considered whether a medical faculty has a written COI policy, all medical schools should get the lowest grade. However, our Internet research and personal contacts with teachers also allowed us to identify courses dealing with COI at several institutions. This is a small but important step, as it allows students to reflect on the ethical choices they are likely to be confronted with once they are in professional practice and to understand them within a broader context. However, these were usually optional elective courses. We scored the policy for medical schools, but listed this score in parentheses as no written documentation was provided for this policy, and it was not listed on the faculty’s website.

In France, as explained by the representative of the Dean’s office of ***Angers***, industry financing is a “delicate issue” that is not discussed. It is possible that this delicacy comes from the fact that part of medical schools’ funding may come from the pharmaceutical industry, such as financial ties between high ranking faculty members and pharmaceutical companies. However, Etain et al. [30] found that faculty members are not opposed to declaring ties with the industry. Other studies have found that students would like more instruction on COI [7,13]. In response to the representative of the Dean’s office of the ***Toulouse Purpan Medical School***, who disapproved of our student-level survey, we note that the U.S. Institute of Medicine (IOM) has recommended action to promote better COI policies by different organizations [31]: “A range of organizations–public and private–can promote the adoption and implementation of conflict of interests policies and help create a culture of accountability that sustains professional norms and promotes public confidence in professional judgments. Institutions that carry out medical research, medical education, clinical care, and practice guideline development have the primary responsibility for addressing conflicts of interest in these activities” (p 29). Although student groups are not explicitly listed, students clearly have a stake in the policies of the institutions where they study, and AMSA has played an important role in policy development in the US.

Three studies with a similar methodology to the one used for our work allow us to do an international comparison of the medical schools' COI policies. These studies were carried out in Australia, the U.S. and Canada.

The Australian study found that 7 medical schools out of 20 had a COI policy, and the Canadian study that 16 of 17 medical schools had a COI [2–3]. This is very different from the French situation, in which only 2 of 37 faculties declared to us that they had any COI policies, but in both cases the policies referred to were informal, rather than a published or written policy.

In 2012 The Pew Charitable Trusts convened a task force to review the literature on COI policies and to make additional recommendations based on the research evidence. In some cases, its recommendations are more restrictive than the ones the AAMC adopted, for example a recommendation to prohibit pharmaceutical sales visits in clinical settings [32].

The delay in France may be partly explained by the lack of action or official recommendations by government or other public bodies concerning COI and medical education. A report by the Assemblée Nationale Social Affairs Committee has recommended action in this area, to “…strive against companies’ interference in future doctors training, especially prohibiting sponsoring or lobbying actions” [33]. However, this recommendation has had no active follow-up.

We hope that this study will encourage more attention to this issue by deans and the administration of medical faculties. French medical faculties would not be starting from zero, as they could benefit from reports and recommendations made by their U.S. counterparts–e.g. the AAMC. Pressure in 2015 from the major student association, the French National Medicine Students Association (ANEMF), could provide additional leverage. A recent initiative by a group of French medical students, “La Troupe du rire” to produce an educational booklet for students on COI and interactions with industry, is indicative of broader interest in policy change [34].

If COI policies are not developed voluntarily, regulatory authorities could take action, such as the National Council for Universities, the Ministry of Health or the State Secretary for Universities and Research.

Furthermore, another approach to shifting the status quo at medical faculties would be to begin with teaching hospitals, as some have shown greater willingness to implement COI policies. For example, l’Assistance Publique—Les Hôpitaux de Paris (AP-HP), the most prestigious French teaching hospital, issued a report about the problem of COI and industry influences in the hospital in March 2016 [35].

Finally, our study results may lead medical schools to take positive action because of publicity about the lack of COI policies. In the U.S., as noted above, the annual AMSA survey contributed to a constant improvement in medical schools’ policies since its initiation in 2007 [32].

In spite of the limited attention to COI at medical faculties reflected in our study, there have been some important policy shifts regulating relationships between medical professionals and industry in France.

The 2003–303 law, introduced on 4 March 2002 regulates the links between medical professionnals and pharmaceutical companies, prohibiting receipt of any in-kind or cash benefit, except in the context of scientific research. The latter must be approved by a professional council. Physicians who are on the staff of medical faculties are among those covered by these provisions. Recent legislation also requires professors to declare conflicts of interest at the beginning of each course or lecture [11]. Not enough time has elapsed, however, to assess compliance in practice.

In France, there is a public database covering industry payments to physicians (www.transparence.sante.gouv.fr) including gifts and meals. The database indicates that from January 2012 to June 2014, French students and residents received gifts worth a total of € 3.96 million [36]. This shows that despite regulations limiting provision of gifts, this practice is still occurring including gifts to medical students and residents. This is highly relevant to the current study, as such gifts become normalized in medical training in the absence of explicit policies restricting or prohibiting them. This is highlighted by survey results indicating that many French medical students fail to see gifts or meals from industry as problematic [12]. The amount received by doctors in practice is much greater: in 2014, 84% of them received at least one payment, amounting to a total of € 172 million [36].

Our study had several limitations, chiefly the low response rate obtained from deans of medical faculties. As the COI policies reported by two deans did not take the form of a written document, we may have missed additional unwritten policies in other medical faculties. However, an unwritten and unpublished policy is unlikely to have the weight of an official policy, and may also be unrecognized by academic staff, increasing the likelihood of violations.

We excluded two criteria in the Shnier et al. study [3], scholarships and free samples, because of expert advice that such practices were rare or non-existent in France. Further investigation is needed to confirm this. It appears that French students are sometimes exposed to samples in teaching hospitals [37]; any further research on COI policies in teaching hospitals will include this criterion.

In conclusion, the AAMC and others organizations have called for a profound cultural change in the medical profession that must begin with medical education [32]. This is all the more important in that students who have the most contacts with industry are also those who tend to have the most positive attitudes to these relations and to believe themselves to be invulnerable to influence [3]. Policies that restrict interactions contributing to conflict of interest have been shown to reduce industry influence on practice [22,23]. Medical faculties in France have a responsibility to protect and educate their students and to support the best possible care, in the interests of patient and public health.

## Supporting Information

S1 File Grading System for Categories in Policies. (DOC)

S1 Table List of the French medical schools and the Internet sites used for the web research. (DOC)
