# Supplementary material for: Conflict of Interest Policies at French Medical Schools: Starting from the Bottom
Source: PLoS One. 2017 Jan 9;12(1):e0168258. doi: 10.1371/journal.pone.0168258 (PMC5221756; doi:10.1371/journal.pone.0168258)
Supplement: S1 File — (DOC) [file pone.0168258.s001.doc]

**S1 File : Grading System for Categories in Policies**

(adapted from Shnier A, Lexchin J, Mintzes B, Jutel A, Holloway K (2013) Too Few, Too Weak: Conflict of Interest Policies at Canadian Medical Schools. PLoS ONE 8(7): e68633.)

**1. Gifts (including meals)**

2 = All gifts and on-site meals funded by industry are prohibited, regardless of nature or value.

1 = Less stringent limitation on industry-funded gifts (e.g., gifts prohibited above €50/year – or gifts prohibited but meals allowed)

0 = No policy, or policy that would not substantially reduce gifting (e.g., gifts are allowed but discouraged, or limited in a non-specific way to “appropriate,” or primarily for the benefit of patients).

**2. Consulting relationships (excluding scientific research and speaking)**

2 = Consulting relationships with industry must be subjected to institutional review or approval. Additionally, they must either be described in a formal contract, or payment for services must be commensurate to the task.

1 = As above, without the institutional review or approval requirement.

0 = No policy, or policy that would allow consulting relationships to occur without institutional scrutiny or that would allow relationships in which payments are not commensurate with work.

**3. Industry-funded speaking relationships/speakers’ bureaus**

2 = Speaking relationships are prevented from functioning as *de facto* gifts or marketing. An effective policy must not implicitly permit (a) long-term speaking agreements or (b) industry to have a role in determining presentation content. (Some effective policies may explicitly prohibit participation in a speakers’ bureau. Other effective policies contain elements such as limits on compensation and reimbursement and a requirement to ensure the scientific integrity of information presented.)

1 = Industry-funded speaking relationships are regulated, but with less stringent limits on longevity, content or compensation.

0 = No policy, or policy that does not define the limits on longevity, content or compensation.

**4. Honoraria**

2 = No acceptance of honoraria; compensation must be at fair market value and publicly disclosed

1 = Limits on accepting/disclosing honoraria.

0 = No policy, or no limits on acceptance.

**5. Ghostwriting**

2 = Ghostwriting is not permitted.

1 = Few or no restrictions; management is left to individual discretion.

0 = No policy.

**6. Disclosure**

2 = No conflict of interests accepted for faculty members, (excepted when there is no other alternative)

1 = Personnel are required to disclose past and present financial ties with industry (e.g., consulting and speaking agreements, research grants) on a publicly-available website and/or disclose such relationships to students when such a relationship might represent an apparent conflict of interest.

0 = No policy.

**7. Industry Sales Representatives**

2 = Pharmaceutical and device representatives are not allowed to meet with faculty regardless of location, or are not permitted to market their products anywhere inside the medical center and associated clinics and offices. (Exceptions may be made for non-marketing purposes, such as training on devices or equipment.)

1 = Pharmaceutical representatives are permitted to meet with faculty, but with significant limitations (e.g., only in non-patient care areas or only by appointment). Exceptions as above.

0 = No policy, or policy that does not substantially limit access.

**8. On-site Education Activities**

2 = Industry is not permitted to provide direct financial support for educational activities, including Continuing Medical Education (CME), directly or through a subsidiary agency.  (However, companies may contribute unrestricted funds to a central fund or oversight body at the academic medical center, which, in turn, would pool and disburse funds for programs that are independent of any industry input or control.)

1 = Less stringent limitations to ensure independence of educational content (e.g., standards to establish freedom from industry influence of content, such as review and approval of presentations; language that prevents industry from selecting the speaker; or language such as: industry funding may be allocated for a particular topic, but must be provided directly to the department, not to individuals).

0 = No policy, or a policy that would not substantially limit industry influence over educational activities (e.g., industry funding must be disclosed).

**9. Compensation for Travel or Attendance at Off-site Lectures & Meetings**

2 = Personnel may not accept payment, gifts or financial support from industry to attend lectures and meetings. (An exception may be made for modest meals, if part of a larger program.) Travel support may only be accepted if it is subject to institutional approval or industry is prevented from selecting (“earmarking”) the recipients. Note: speaking and consulting relationships are evaluated separately in domain 1.

1 = Less stringent limitations.

0 = No policy, or a policy that would not substantially limit participation in industry-funded events and meetings.

**1O. Medical school curriculum (or other documentation of educational objectives/course content)**

2 = Students are trained to understand institutional conflict-of-interest policies and recognize how industry promotion can influence clinical judgment.

1 = Curriculum addresses conflict of interest in a more limited way (e.g., training on policies only).

0 = No policy (not addressed in curriculum or elsewhere).

**11. Pharmaceutical industry funding of the medical school.**

2 = No funding accepted

1 = Funding accepted, but a limited fixed amount, and with a special attention so that this funding do not interfere with the mission of the medical school to educate and protect its students from the pharmaceutical influences.

0 = No policy, or policy that does not substantially limit access.

**12. Industry educational support of residents for publication of scientific articles.**

2 = The Medical Faculty does not accept publication assistance from pharmaceutical companies for its students ; or faculty are told to inform their students about the backgrounds and potential consequences for accepting this « help » provided by the pharmaceutical firms. The medical school commits itself to provide an adequate formation in this field to their students.

1 = Less stringent policy

0 = No policy

**13. Medical school activities to promote COI policies in affiliated teaching hospitals.**

2 = Active policy to encourage other training places, especially teaching hospitals, to respect and follow the medical school COI policy.

1 = Less stringent policy

0 = No policy

**14. Enforcement**

A. Is it clear that there is a party responsible for general oversight to ensure compliance?  (Y/N)

B. Is it clear there are sanctions for noncompliance?  (Y/N)
