# Supplementary material for: Conflict of Interest Policies at French Medical Schools: Starting from the Bottom
Source: PLoS One. 2017 Jan 9;12(1):e0168258. doi: 10.1371/journal.pone.0168258 (PMC5221756; doi:10.1371/journal.pone.0168258)
Supplement: S1 Table — (DOC) [file pone.0168258.s002.doc]

**S1 Table : List of the French medical schools and the Internet sites used for the web research**

| Medical school | Web site |
| --- | --- |
| AMIENS | <http://www.u-picardie.fr/jsp/fiche_structure.jsp?STNAV=&RUBNAV=&CODE=UI&LANGUE=0> |
| ANGERS | <http://www.med.univ-angers.fr/> |
| BESANÇON | <http://medecine-pharmacie.univ-fcomte.fr/> |
| BORDEAUX | <http://www.u-bordeaux2.fr/M-00-/0/fiche___defaultstructureksup/> |
| BREST | <http://www.univ-brest.fr/medecine/> |
| CAEN | <http://ufrmedecine.unicaen.fr/> |
| CLERMONT-FERRAND | <http://medecine.u-clermont1.fr/> |
| DIJON | <http://medecine.u-bourgogne.fr/> |
| GRENOBLE - LA TRONCHE | <http://www-sante.ujf-grenoble.fr/SANTE/cms/sites/medatice/home/portail/index.php?pid=85> |
| GUADELOUPE | <http://www.univ-ag.fr/fr/institution/u_f_r/ufr_medecine.html> |
| LA RÉUNION | <http://ufr-sante.univ-reunion.fr/> |
| LILLE 2 | <http://medecine.univ-lille2.fr/> |
| LILLE - Université Catholique de Lille | <http://flm.icl-lille.fr/index.asp> |
| LIMOGES | <http://www.medecine.unilim.fr/scolarite/> |
| LYON ESt | [http://lyon-est.univ-lyon1.fr](http://lyon-est.univ-lyon1.fr/) |
| LYON – OULLINS | [http://lyon-sud.univ-lyon1.fr](http://lyon-sud.univ-lyon1.fr/) |
| MARSEILLE | [http://medecine.univ-amu.fr](http://medecine.univ-amu.fr/) |
| MONTPELLIER | <http://www.med.univ-montp1.fr/> |
| NANCY | <http://www.medecine.univ-lorraine.fr/> |
| NANTES | <http://www.medecine.univ-nantes.fr/> |
| NICE | <http://medecine.unice.fr/> |
| PARIS V – DESCARTES | <http://www.medecine.parisdescartes.fr/> |
| PARIS VI - PIERRE ET MARIE CURIE | <http://www.medecine.upmc.fr/> |
| PARIS VII – DIDEROT | <http://www.medecine.univ-paris-diderot.fr/> |
| PARIS XI - LE KREMLIN BICÊTRE | <http://www.medecine.u-psud.fr/fr/index.html;jsessionid=DCDFF22D1DB003C2B9693DBBDF789D56.ufrmedecine> |
| PARIS XII – CRÉTEIL | <http://medecine.u-pec.fr/> |
| PARIS XIII – BOBIGNY | <http://www-smbh.univ-paris13.fr/> |
| PARIS-IDF OUEST - MONTIGNY LE BRETONNEUX | <http://www.medecine.uvsq.fr/> |
| POITIERS | <http://medphar.univ-poitiers.fr/> |
| REIMS | <http://www.univ-reims.fr/formation/formation/ufr-instituts-et-ecoles/ufr-de-medecine/actualitert_id=> |
| RENNES | <http://www.medecine.univ-rennes1.fr/> |
| ROUEN | <http://medecine-pharmacie.univ-rouen.fr/> |
| SAINT ÉTIENNE | <http://portail.univ-st-etienne.fr/bienvenue/presentation/ufr-de-medecine-300420.kjsp?RH=0701261013> |
| STRASBOURG | <http://medecine.unistra.fr/> |
| TOULOUSE Purpan | <http://www.medecine.ups-tlse.fr/index.php> |
| TOULOUSE Rangueil | <http://www.medecine.ups-tlse.fr/index.php> |
| TOURS | <http://www.med.univ-tours.fr/?idItem=1> |
